# Engaging Cancer Care Physicians in Off-Label Drug Clinical Trials: Human-Centered Design Approach

**DOI:** 10.2196/51604

**Published:** 2024-02-15

**Authors:** Maren C Parsell, Morgan N Greenleaf, Greeshma G Kombara, Vikas P Sukhatme, Wilbur A Lam

**Affiliations:** 1 Georgia Clinical and Translational Science Alliance Emory University School of Medicine Emory University Atlanta, GA United States; 2 Emory School of Medicine Emory University Atlanta, GA United States; 3 Center for the Advancement of Diagnostics for a Just Society (ADJUST) Emory University Atlanta, GA United States; 4 The Morningside Center for Innovative and Affordable Medicine Emory University School of Medicine Emory University Atlanta, GA United States; 5 Departments of Medicine and Hematology and Medical Oncology Emory University Atlanta, GA United States; 6 Aflac Cancer and Blood Disorders Center of Children’s Healthcare of Atlanta and Department of Pediatrics Emory University Atlanta, GA United States

**Keywords:** human-centered design, clinical trial design, design methods, clinical trial, trial methodology, barriers, off-label drugs, stakeholders, cancer, medications

## Abstract

**Background:**

Using a human-centered design (HCD) approach can provide clinical trial design teams with a better understanding of the needs, preferences, and attitudes of clinical trial stakeholders. It can also be used to understand the challenges and barriers physician stakeholders face in initiating and completing clinical trials, especially for using off-label drugs (OLDs) to treat unmet clinical needs in cancer treatment. However, the HCD approach is not commonly taught in the context of clinical trial design, and few step-by-step guides similar to this study are available to demonstrate its application.

**Objective:**

This study aims to demonstrate the feasibility and process of applying an HCD approach to creating clinical trial support resources for physician stakeholders to overcome barriers to pursuing clinical trials for OLDs to treat cancer.

**Methods:**

An HCD approach was used to develop OLD clinical trial support concepts. In total, 45 cancer care physicians were contacted, of which 15 participated in semistructured interviews to identify barriers to prescribing OLDs or participating in cancer OLD clinical trials. Design research is qualitative—it seeks to answer “why” and “how” questions; thus, a sample size of 15 was sufficient to provide insight saturation to address the design problem. The team used affinity mapping and thematic analysis of qualitative data gathered from the interviews to inform subsequent web-based co-design sessions, which included creative matrix exercises and voting to refine and prioritize the ideas used in the final 3 recommended concepts.

**Results:**

The findings demonstrate the potential of HCD methods to uncover important insights into the barriers physicians face in participating in OLD clinical trials or prescribing OLDs, such as recruitment challenges, low willingness to prescribe without clinical data, and stigma. Notably, only palliative care participants self-identified as “frequent prescribers” of OLDs, despite high national OLD prescription rates among patients with cancer. Participants found the HCD approach engaging, with 60% (9/15) completing this study; scheduling conflicts caused most of the dropouts. Over 150 ideas were generated in 3 co-design sessions, with the groups voting on 15 priority ideas that the design team then refined into 3 final recommendations, especially focused on increasing the participation of physicians in OLD clinical trials.

**Conclusions:**

Using participatory HCD methods, we delivered 3 concepts for clinical trial support resources to help physician stakeholders overcome barriers to pursuing clinical trials for OLDs to treat cancer. Overall, integrating the HCD approach can aid in identifying important stakeholders, such as prescribing physicians; facilitating their engagement; and incorporating their perspectives and needs into the solution design process. This paper highlights the process, methods, and potential of HCD to improve cancer clinical trial design. Future work is needed to train clinical trial designers in the HCD approach and encourage adoption in the field.

## Introduction

### Project Goals

Cancer clinical trials involve the coordination of many stakeholders but frequently fail to meet enrollment goals, prespecified end points, and timelines [1,2]. Effective stakeholder engagement can be essential to cancer clinical trial design, conduct, and reporting of clinical research [3]. However, traditional design approaches often lack consideration of stakeholders’ needs, preferences, and experiences, which can lead to recruitment and retention challenges or to study results that are less generalizable or applicable to real-world settings. Trial stakeholders include physicians who face unique barriers, especially when designing trials for off-label drugs (OLDs) to treat unmet clinical needs in cancer treatment [4]. Assumptions that stakeholders will be willing to participate in a trial may not adequately factor in the unique challenges and barriers that patients with cancer and stakeholders face. In addition, underrepresented and marginalized populations’ needs can also be missed because their perspective is rarely represented by clinical trial designers, which can lead to a lack of diversity in clinical trial participants and potential disparities in treatment outcomes [1].

To overcome these limitations, a human-centered design (HCD) approach offers both a process to learn about the needs of the trial stakeholders as well as flexible tools to test assumptions, uncover barriers, and work collaboratively with study stakeholders to optimize the clinical trial design. An emerging body of literature on HCD for health care innovation yields many HCD methods to choose from [5], making it difficult for novice innovators to know where to start and which methods to use. The purpose of this paper is to demonstrate HCD methods through a case study, where cancer care physicians participated in developing solutions that reduced barriers associated with initiating clinical trials for promising OLDs.

### Background

HCD is a problem-solving methodology that focuses on discovering needs and developing solutions for individuals within a system; it is increasingly being used in cancer care settings [6-8]. HCD is particularly well-suited for uncovering and understanding the complex attitudes and barriers contributing to physician off-label prescribing behavior for a variety of Food and Drug Administration–approved drugs with strong safety profiles such as aspirin that may have anticancer properties [9]. These medications have shown early evidence when prescribed off-label, in reducing the risk of developing certain types of cancer such as colorectal cancer, or in improving patient outcomes. However, due to low financial incentives and resources, and the lack of further clinical testing to provide definitive prospective data, physician awareness is low, and prescription adoption has been minimal [10]. To help build incentives and engage physicians to consider repurposed OLD options, the Morningside Center for Innovative and Affordable Medicine (Morningside Center) [11] based at Emory University worked with staff trained in HCD methods from the Innovation Catalyst program within the Georgia Clinical and Translational Science Alliance (CTSA) [12]. The Georgia CTSA team set out to first learn more about the barriers to these types of prescriptions and then work together with physicians to propose support programs to increase the consideration of repurposed OLD treatment options for their patients. We explored the implications of these findings for physician receptiveness to conducting clinical trials with repurposed OLDs.

## Methods

### Study Design

**Figure 1:**
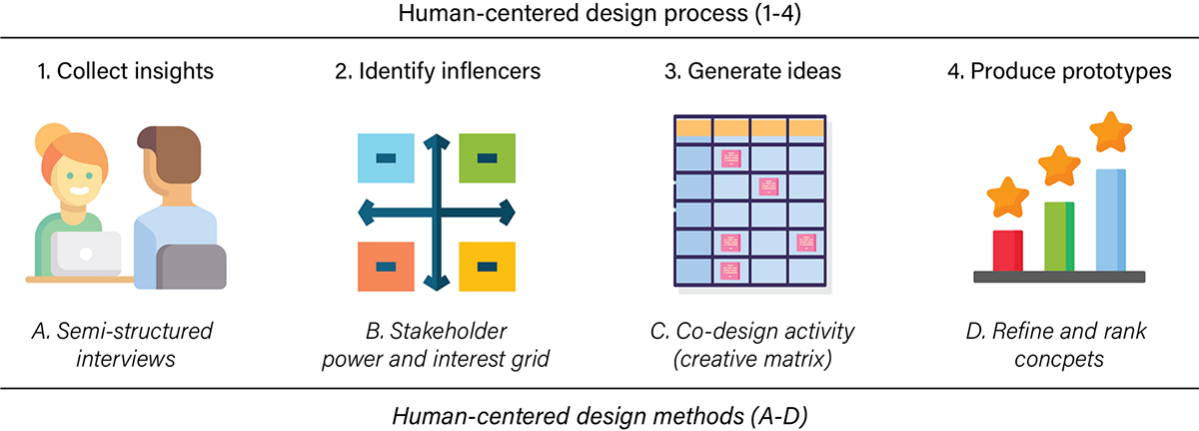
Illustration of human-centered design process and methods used in this study.

HCD is an iterative and flexible process for generating solutions to problems that typically includes 3 phases: learning about the humans involved in the situation, coming up with and refining concepts, and implementation. Additional steps can be added to address the specific needs of the project, and the CTSA research team emphasized stakeholder analysis because many stakeholders influence prescription decisions, so understanding who had the greatest influence was important to forming the guiding questions for the ideation activity. Thus, the team followed four steps with each step matched with an appropriate HCD method: (1) collect, sort, and analyze insights using semistructured interviews and affinity mapping; (2) identify influences using a stakeholder power-interest grid; (3) generate and prioritize ideas using a co-design activity called a “creative matrix” [13]; and (4) produce concepts to prototype by refining and ranking ideas generated from the co-design activity (Figure 1). The team identified 45 physicians who met the cancer care inclusion criteria within the Emory academic medical center ecosystem or community practice setting. From this pool, 15 agreed to participate in semistructured interviews. Of the 15 physicians interviewed, 8 were oncologists, 3 were palliative care specialists, 2 were urologists, 1 was an anesthesiologist, and 1 was a family medicine physician (Multimedia Appendix 1). Although this sample size is small, it was adequate to achieve insight saturation, or the point where participants were providing similarly themed insights.

### Collect Insights

Within the HCD approach, qualitative research methods such as interviews, focus groups, or ethnography are typically used to gain insights about the situation, influencing factors, as well as the needs and wants of the stakeholders involved. The Georgia CTSA research team conducted semistructured interviews, which involve asking open-ended questions that allow for more free-flowing conversation than in structured interviews. A set of predetermined questions or topics guided the conversation, but there was also flexibility to follow-up on interesting or unexpected responses and to explore topics in more detail. Some of the key questions the team sought to answer were:

- Have you prescribed OLDs? Why or why not?
- What level of information or data do you need to prescribe?
- Where do you find information on OLDs?
- Who influences your OLD prescription decisions?
- What influences your decision to participate in OLD clinical trials?

**Figure 2:**
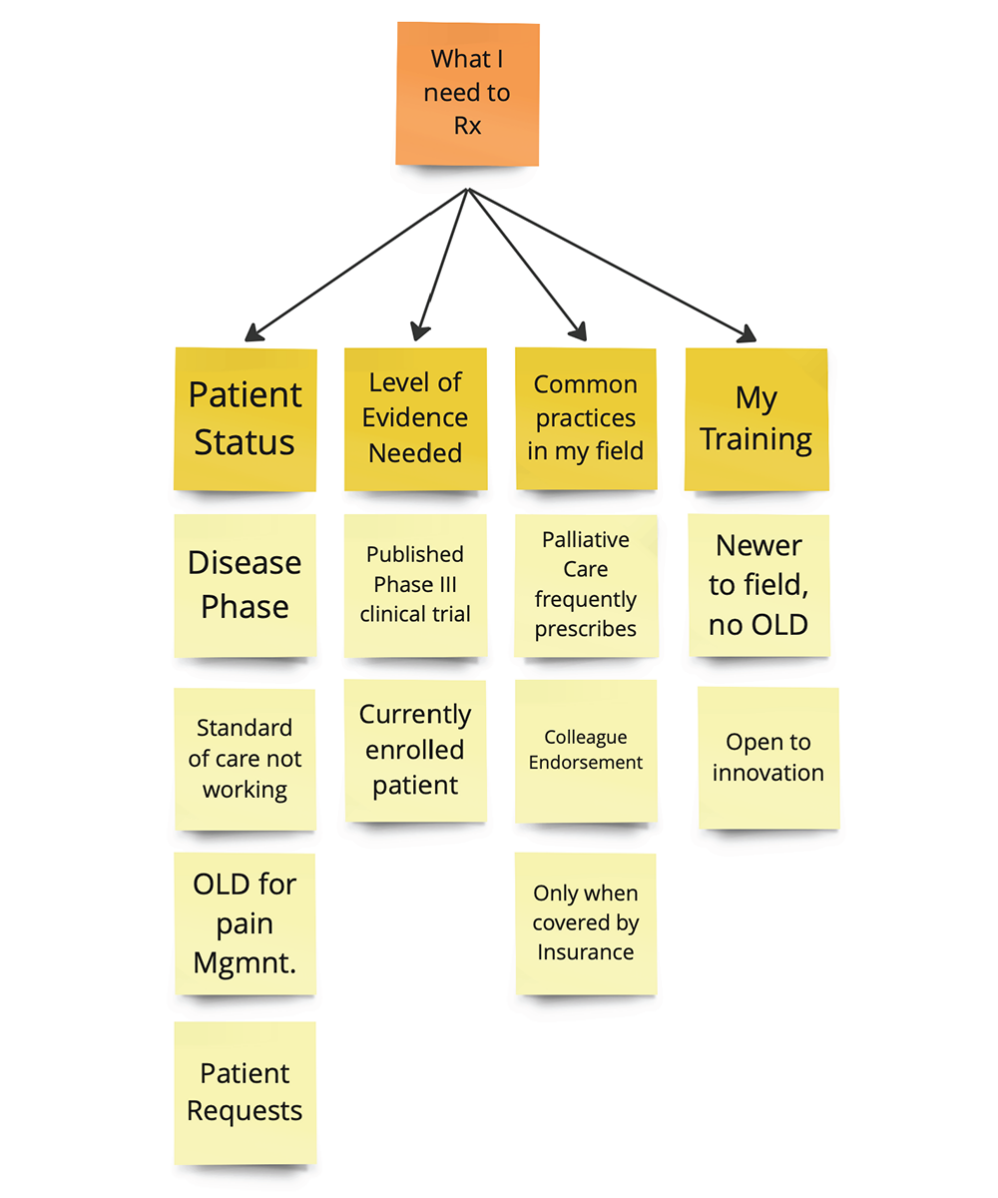
Screenshot of affinity mapping method used for sorting insights in this study. Mgmnt: management; OLD: off-label drug; Rx: doctor's prescription.

The interviews produced dozens of pages of transcribed data, which the team reduced to key points on Post-it notes in preparation for a thematic analysis process called affinity mapping. Concepts that are related to each other were grouped, and a theme was coded for each group (Figure 2). In addition, stakeholders who influenced prescription decisions were also noted.

### Identify Influencers

**Figure 3:**
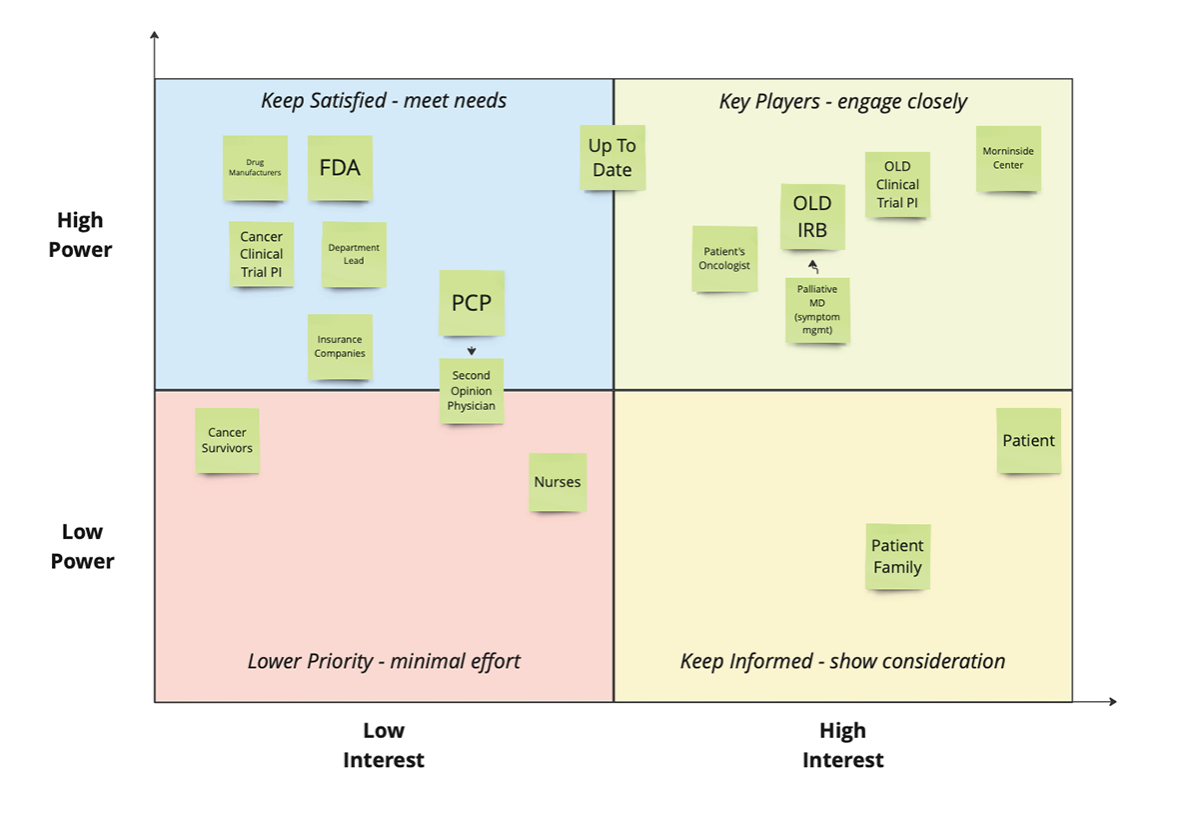
Screenshot of power-interest grid method used for stakeholder analysis in this study. FDA: Food and Drug Administration; IRB: institutional review board; MD: Doctor of Medicine; mgmt: management; OLD: off-label drug; PCP: phencyclidine; PI: principal investigator.

To validate assumptions about who ultimately makes OLD prescribing decisions and to verify decision-making impact, the Georgia CTSA research team identified stakeholders based on the insights collected from the interviews and affinity mapping process (Multimedia Appendix 2). This list of stakeholders was then mapped to a power-interest grid, which involves plotting stakeholders on a 3D grid based on their level of power or influence over the situation and their level of interest in outcomes (Figure 3). Stakeholders with high levels of power and high levels of interest are considered key players and require the most engagement while high-interest and lower-power stakeholders should be kept updated.

### Generate Ideas

**Figure 4:**
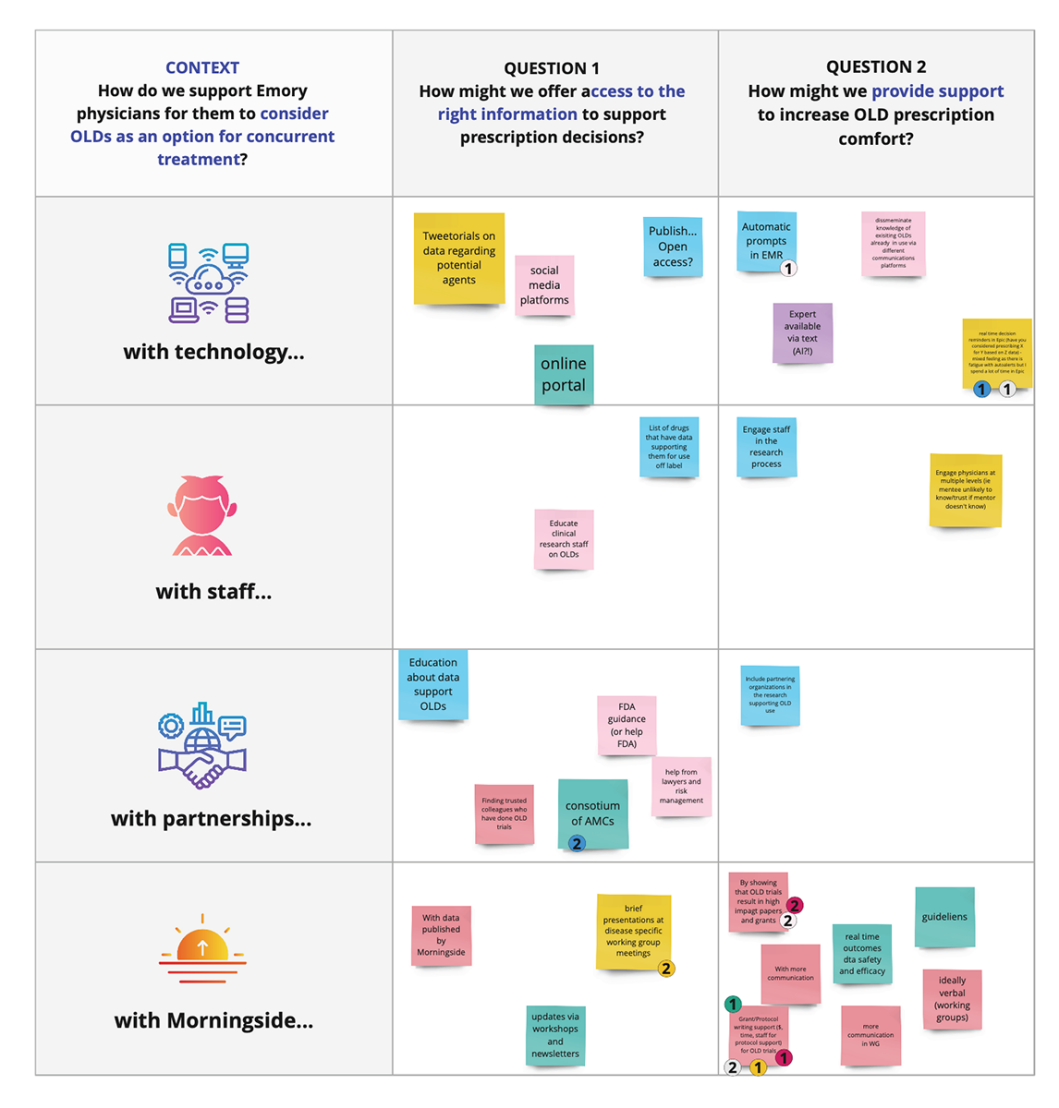
Screenshot of creative matrix method of guided ideation used in this study. AI: artificial intelligence; AMC: academic medical center; EMR: electronic medical record; FDA: Food and Drug Administration; OLD: off-label drug; WG: working group.

Co-design activities include the end users of a solution in the ideation of that solution. Co-design is an effective way to increase stakeholder engagement, which is essential to project success, particularly in cancer care delivery research [3]. There are many methods to collaboratively come up with ideas, such as rapid ideation, brainwriting, mind mapping, thumbnail sketching, and many others. Which method to select depends on the goals of the session, the time allotted, and who is participating. The Georgia CTSA team selected the creative matrix method (Figure 4), which concentrates ideation on a particular topic using a visual grid with 2 questions and up to 4 categories. This method can generate a high volume of ideas in a short time and within a specific context. The research team selected salient topics that arose from the affinity mapping exercise to create the context for 2 creative matrix exercises used in three 1-hour co-design sessions with 9 physicians. Participants were allotted 6 minutes to generate ideas and 3 minutes to select 2 concepts that they believed had the most valuable to help support the consideration of repurposed OLDs for prescription. These “best ideas” were subsequently mapped on a grid for impact versus effort to identify which ideas would have the most impact and lowest effort.

### Vote and Produce Prototypes

Involving co-design participants in the idea evaluation process is important for getting feedback on the feasibility, desirability, and viability of the ideas [14]. It can increase participants’ sense of ownership and investment in the final solution, potentially leading to greater adoption and success. While co-design sessions can generate a high volume of ideas quickly, further refinement to create workable prototypes for testing is sometimes necessary, and this study’s team distilled concepts that were well-received by physicians during the co-design sessions into 3 prototype-ready directions.

### Ethical Considerations

This study was reviewed by an ethics committee to ensure the protection of research participants, and it was approved by Emory University Institutional Review Board (STUDY00004025). Each participant provided consent to be interviewed and that their responses could be used in our design study without additional consent. The privacy and confidentiality of participants and their responses were ensured by removing names and any other fact that might point to participants’ identity in this study; also, research records are kept private to the extent required by law. The compensation for participation in this study was a US $50 gift card.

## Results

### Thematic Analysis

Of the 45 physicians we contacted, 15 agreed to be interviewed and share their experiences and opinions on OLD prescribing for cancer care. Key themes that emerged from the interviews showed that, in contrast to documented high off-label prescription rates in cancer [15-17], the interviewed physicians were less likely to self-identify as “frequent prescribers,” citing a range of barriers. A lack of clinical trial evidence to support the prescription was most frequently expressed as a barrier to prescription, as well as cost and reimbursement concerns and the lack of knowledge of off-label options. A notable exception to off-label prescription hesitancy was seen in palliative care specialists who expressed few barriers to prescribing off-label but emphasized that their focus was on easing symptoms and not providing treatment. To learn about OLDs, physicians described a variety of sources but most often relied on professional social networks to discover and deepen knowledge; however, they struggled to find information on specific OLDs when they needed it.

On the power-influence grid, cancer care physicians were identified as the stakeholders having the highest power and interest in patient prescriptions, validating the assumption that they are the primary prescription decision makers who require the most engagement. Patients and their families were mapped to the high-interest but less power quadrant and were sometimes the source of OLD prescription requests that did not always lead to prescriptions.

### Concept Generation

The Georgia CTSA research team synthesized insights from the interviews into 2 brainstorming themes used during 3 co-design sessions with 9 physician participants. The first context explored types of support needed for physicians to consider OLDs as concurrent treatment, which generated 70 concepts. The second context focused on what support was needed to make it easier to start and complete an OLD clinical trial, which produced 95 concepts. Of the 165 total concepts, participants voted for 14 of the ideas that they felt best supported their needs, which the physicians mapped to identify which were high impact or lower effort. The selected concepts included providing dedicated clinical research coordinators, protected time for clinical trials, electronic medical record prompts or reminders, incentive programs, an OLD reference database or app, and endorsements for OLD trials at team meetings to support recruiting efforts (Multimedia Appendix 3).

The research team further synthesized these concepts into 3 prototype-ready directions listed in Textbox 1.

**Recommended concepts delivered to the Morningside Center after this study.**

**Concept and description**

- Off-label drug (OLD) fellowship program
  An invitation-only program for physicians initiating OLD clinical trials that emphasizes promotional communications for the principal investigator (PI) to promote the study and offers protected research time.
- OLD clinical trial support package
  Sponsored package of select administrative resources including a clinical resource coordinator to facilitate OLD trials.
- OLD searchable database
  On-demand digital tool to provide national repurposed OLD updates, identify center-wide OLD trials and PIs, and look up available clinical trial resources.

**Figure 5:**
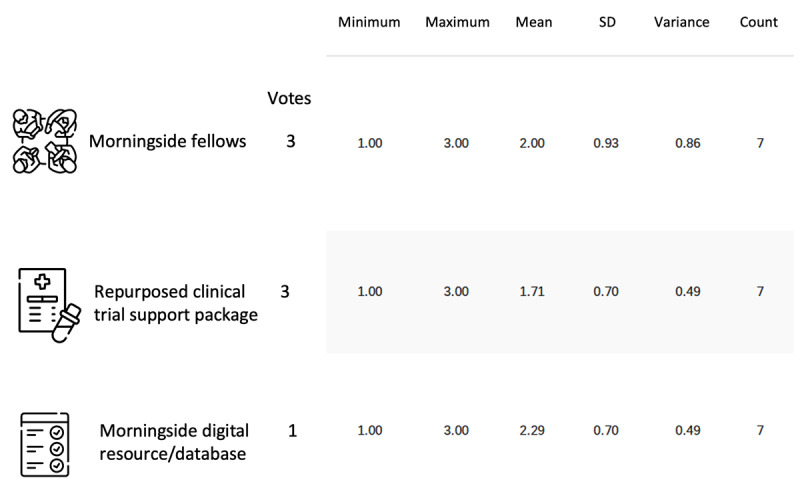
Force ranked physician votes for 3 prototype-ready concepts to support repurposed OLD prescription decisions. OLD: off-label drug.

In a web-based survey, all 15 physician participants were asked to force rank the concepts that they felt was the most impactful to supporting repurposed OLD prescription decisions; we received 7 responses (Multimedia Appendix 4). The fellowship program concept surfaced as the leading idea with a mean of 2.0 (SD 0.93; Figure 5). Overall, concepts that resonated most either supported clinical trial development or bolstered social interfaces from which they could learn about OLDs from other physicians.

## Discussion

### Principal Findings

Using this case study, we have demonstrated the flexible nature of HCD as it relates to accommodating unique characteristics of the project, selecting from a wide range of ideation techniques, and adapting to participant ideas and preferences. The findings also demonstrate the potential of HCD methods to uncover important insights into physician decision-making processes that can lead to improving OLD prescription and clinical trial practices. Clinical trial designers can similarly use these methods to gain a deeper understanding of the target audience’s needs, preferences, and attitudes toward a potential trial. Insights can help overcome barriers to participation, such as language or cultural differences, and inform more effective trial protocols that lead to greater participant engagement and retention. Without using the grounded approach of first synthesizing stakeholder insights, ideation sessions may not be as productive.

A notable feature of HCD is high engagement among participants, also demonstrated in this study with 60% (9/15) of physicians participating from the initial interviews to the ideation and final voting exercises. Further, 1 participant said, “I really appreciate being listened to and would be willing to volunteer to test these ideas.” HCD can be used to increase patient engagement by incorporating the needs, preferences, and attitudes of potential trial participants [18]. Participants who did not participate in all activities of this study either did not respond to the invitations or declined because of scheduling conflicts.

HCD’s participatory nature contrasts with the traditional clinical trial design, which often does not engage the participants for which the trial is designed. This can lead to important perspectives or data being missed and result in low patient engagement and incomplete or biased research outcomes. The high failure rate of clinical trials has led to increasing interest in using stakeholder and patient engagement to support clinical trial design [3,19]. In the context of clinical trial design, a co-design activity could involve collaborating with patients, health care providers, and other stakeholders to co-design trial materials, such as patient education materials or data collection forms to ensure that they are acceptable and feasible.

While HCD offers a flexible process and many methods to choose from, it is very different from the linear thinking and hierarchical norms associated with traditional scientific approaches [20], which may thwart the adoption or effective implementation of the HCD process. Additional challenges can include the potential for interviewer bias and the time- and resource-intensive nature of conducting interviews and co-design workshops. While HCD is becoming an increasingly familiar term, access to training remains low as few institutions have internal design teams, usually found in innovation centers [21], to consult with or to run projects. Thus, acquiring and building HCD expertise can be expensive, and it remains to be seen how future clinical innovation funding or such services will be allocated.

### Conclusions

Although a relatively new problem-solving approach in health care, HCD can provide tangible, flexible, and reproducible methods that include stakeholders in the ideation and problem-solving process [22]. HCD can help identify and engage important stakeholders such as physicians and patients in the design process and then integrate their perspectives and needs into the subsequent solutions, which is increasingly seen as an important contributor to cancer clinical trial success. This case study provides a step-by-step guide on how to apply the HCD process and selected methods to generate stakeholder-centered solutions. HCD has the potential to be an important tool to increase success rates of clinical trials, but increased institutional support and researcher training will be needed for HCD to provide its fullest benefit.

## Appendix

Multimedia Appendix 1 List of physicians who participated in the study, their specialties, and co-design session attendance status.

Multimedia Appendix 2 Brainstorm activity to record all stakeholders who influence the prescption of off-label drugs, used as the first step in the stakeholder mapping exercise.

Multimedia Appendix 3 Co-design activity concepts organized by innovation question.

Multimedia Appendix 4 Descriptions of prototype concepts with survey results from physician participants.

## Abbreviations

CTSA: Clinical and Translational Science Alliance
HCD: human-centered design
OLD: off-label drug

## Data Availability

All data generated or analyzed during this study are included in this published paper and its supplementary information files.
