# Supplementary material for: Engaging Cancer Care Physicians in Off-Label Drug Clinical Trials: Human-Centered Design Approach
Source: JMIR Form Res. 2024 Feb 15;8:e51604. doi: 10.2196/51604 (PMC10905356; doi:10.2196/51604)
Supplement: Multimedia Appendix 1 [file formative_v8i1e51604_app1.pdf]

Physician Participation in Morningside Off Label Drug Study

Physician	Specialty	Completed Interview	Requires clinical data or a trial to prescribe an OLD	Attended co-design session
1	Oncologist	Y	Y	Y
2	Oncologist	Y	Y	N
3	Oncologist	Y	Y	Y
4	Oncologist	Y	Y	Y
5	Oncologist	Y	Most of time	N
6	Oncologist	Y	Y	Y
7	Palliative Care	Y	Y	Y
8	Palliative Care	Y	N	Y
9	Urologist	Y	N	N
10	Anesthesiologist (Cancer)	Y	N	Y
11	Palliative Care	Y	N	Y
12	Oncologist	Y	Y	N
13	Family and preventative	Y	Most of the time	N
14	Medical Oncologist	Y	Y	Y
15	Medical Oncologist	Y	N	N
