# Supplementary material for: Engaging Cancer Care Physicians in Off-Label Drug Clinical Trials: Human-Centered Design Approach
Source: JMIR Form Res. 2024 Feb 15;8:e51604. doi: 10.2196/51604 (PMC10905356; doi:10.2196/51604)
Supplement: Multimedia Appendix 2 [file formative_v8i1e51604_app2.pdf]

Stakeholder Map

1. Gather All Stakeholders

Gather All Stakeholders

10 min

This stage is to identify all the stakeholders who influence the prescription of off label drugs. These range from direct prescription decisions to influencers of the availability of data and drugs

Healthy oncology patient who is not responding to treatment

Primary Physician (Usually not primary **care** physician)

Medical School professors (establish culture)

Academic medical center clinical trial PIs

FDA (Establish indications)

Community oncologists who have run out of options for a particular patient

Physicians who manage pain (particularly palliative care)

Medical journals (their editors)

Drug mfg - is the drug generic and cheap?

Morningside (as funder/promoter)

Academic centers generally (as places where clinical studies happen)

CROs

Palliative MD

Nurse

Social Media contacts

Patient family

Online Patient forums

Weekly physician panel meeting

Publications

UptoDate

Guidelines

Members of Care team

Oncologist

Disease working group physicians

Insurance company

Peers (Call a friend)

Clinic manager?

Cancer survivors?

Google

Online sources

Pharmacist

"Go To" person "Call a Friend"

Tool Tip

Use the Miro timer in the bottom menu.

Tool Tip

The Bulk Mode tool (part of the Sticky Note feature) can help while brainstorming.

DUPLICATES

Physicians in the same field (What has worked for you?)

Primary Physician

Physician team lead

Nurse

Patient

Patient

IRB

IRB

The people who manage the insurance appeal process?

Insurance! (won't cover if off label) - Who is this specifically? Job title?

Patient Family

NCCI Guidelines

PubMed

Patient care team

1

You're offline. Will try to reconnect in 5 seconds. Try now

37%

?
