# Supplementary material for: Engaging Cancer Care Physicians in Off-Label Drug Clinical Trials: Human-Centered Design Approach
Source: JMIR Form Res. 2024 Feb 15;8:e51604. doi: 10.2196/51604 (PMC10905356; doi:10.2196/51604)
Supplement: Multimedia Appendix 3 [file formative_v8i1e51604_app3.pdf]

Co-Design Concepts

Suggested by physician study participants

How might we **provide support** to increase OLD prescription comfort? *(* = number of votes)*

- **Use different communication platforms to share existing OLD knowledge**
  - Social Media (ex. Tweetorials by docs)
  - Ask questions via text to experts (AI) or hotline
  - Ideal way: Verbally
  - Monthly Newsletters
  - Quarterly Morningside presentations
    - Emphasize "WHY" – why cheaper, why effective, why safe etc. throughout
    - Show that OLD trials = high impact papers and grants **
  - Database App*
    - Has contact info for experts/OLD PIs
    - Provide current data (ex. Real-time reactions to the drugs)
- **Automatic prompts in EMR ***
  - Real time reminders via EPIC **
  - Include DTA safety and efficacy

- **Types of support**
  - Money, time, staff, etc.
  - Legal support
  - Create grant/protocol writing support for OLD trials ****
  - Engage staff in research process
    + multiple levels throughout (Ex. PharmD)
- **Partnerships**
  - Collaborate with organizations that partnered with previous OLD research
  - Create multi-site university teams to support clinical decision making for OLDs
  - Create multi-institutional interest group for OLDS

How might we **offer access** to the right information to support prescription decisions? *(* = number of votes)*

- **Educating clinical research staff on OLD data**
  - Workshops + Newsletter updates through Morningside
  - AMC consortium **
  - Presentations during disease specific group meetings (Morningside specific) **
  - Training
- **Online Portal / OLD Database including supporting data + literature**
  - Include published Morningside Data
  - Create free app with data **
    - App funded by Morningside **
  - Multi-site database could be Emory-based or open to all data
  - Include contact information for local experts
- **Publishing in Open Access Journals**
- **Social media**
  - Tweetorials on data related to new OLDs
- **Connecting colleagues who completed OLD trials**
  - Emory OLD directed contact / peer network
  - Connecting with PIs working on current trials
- **Increasing FDA guidance**
- **Assistance with risk management lawyers**
- **Info in EMR**
  - OLD pop-up options in EPIC
- **Increasing patient awareness**
  - Similar to Pharma ads “ask your doctor about…”
- **Train PharmD to assist with questions**
- **Industry partnership = important**
  - Most OLD drugs no longer exist due to lack of financial incentives

How might we **increase recruitment** of off label drug trials at Emory so that promising data can be validated?

- **Patient level outreach**
  - Social media or sites were patients go to look for enrollment trials **
    - Have Emory groups promote trials
    - More advertising on campus
  - Develop hand-outs for potential candidates
  - Clinic TVs for advertising
    - Provide videos on OLD
  - Tap into condition platforms for patients
    - Partner w online community affected by the disease/disease-based advocacy groups
- **Community/private practice oncologists as primary enrollment source****
- **Digital Platform**
  - Set up platform on OLD success
  - Provide qualitative data on patients that refused to participate **
  - Allow clinicians + pharmacists to use platform to document in real time when using OLD
  - License a platform/service for recruitment
    - Morningside for recruitment/provide CRCs
- **EPIC alerts about candidates for enrollment***
- **Ask pharmacists frequently to identify patients for study**
- **Communications to Physicians**
  - Seminars – PI present open trials
  - Winship based virtual symposium
- **Collaborate with other institutions**
  - Universities; partner w/another AMC on trials, CTSA
  - Cancer organizations/non-profits
  - Industries that can provide these medications
  - Collaborate with key physicians in these fields / champions of these medications
- **Advocate for less bureaucracy around clinical trials – more mainstreamed process**
- **Hire more**
  - Have 1 designated clinical recruiter focused on OLD per department
  - Students/fellows
  - Floating staff
  - Dedicated staff at on-site pre-screen clinics
    - Enough CRCs
- **Ensure that doctors are protected from liability**

How might we **incentivize Emory physicians** to open off-label drug trials? *(* = number of votes)*

- **Financial**
  - Pay members for each accrual ***
  - Financial incentive for docs on per enrollee basis and participation
  - OLD trials come with FTE%
  - Increase philanthropy to provide protected time for researchers to do this research that may not come from Pharma $
    - Local seed funding for PIs to start pilot trials (can be internal funding)
- **Frequent communication between investigators and physicians**
  - Have senior faculty in ECOG talk to ECOG leaders to support OLD trials **
  - Regular meetings to discuss real time data *
  - Can use social media to communicate success between groups
  - Highlight faculty that complete trials
  - Hand-outs w info on importance
- **Staffing Support**
  - More CRCs + people to take care of admin work and legal issues
  - Help w IRB submissions
  - Protocol support/medial writers
- **Technology**
  - Create database
    - App with all active Emory OLD trials
  - Offer technology upgrades
    - Free access to trial-management software
- **Decision support**
  - Streamlined IND process from Emory and Pharma partnership to create drug trials and overall, less barriers for PIs to start research from ground up
  - Pick trials that offer treatment options and not only ask scientific questions

Top-Rated Impact/Difficulty Concepts

Clinical Trial Incentives

- Provide support for OLD trials (money, time, resources)
- Provide CRC support
- Offer protected time/salary support for PI to engage in trial
- Offer a team to support PI in clinical trial, use as an incentive to encourage them to launch an OLD trial

Digital Resource

- Create an app that lists type of illness, links to references and level of evidence
- Database of drugs that have been prescribed off-label at Emory (include info on why is it cheaper, does it work, is it safe, etc)

Communications

- Verbal endorsement/promotion of OLD (at meetings)

Trial Enrollment

- Tap into condition platforms to advertise trials for recruiting
- OLD trial enrollment is 10x more difficult; need more enrollment support
- Pay for recruits to trials (finder's fee)
- Partner with compounding pharmacies and industry to advance clinical trial (compassionate use)
