# Supplementary material for: Engaging Cancer Care Physicians in Off-Label Drug Clinical Trials: Human-Centered Design Approach
Source: JMIR Form Res. 2024 Feb 15;8:e51604. doi: 10.2196/51604 (PMC10905356; doi:10.2196/51604)
Supplement: Multimedia Appendix 4 [file formative_v8i1e51604_app4.pdf]

3 Prototype Concepts

Voted on by physician study participants

Final Concepts Ranking Questionnaire

EMORY UNIVERSITY SCHOOL OF MEDICINE

The Morningside Center for Innovative and Affordable Medicine (Morningside Center) was created to address the lack of financial incentive that inhibits promotion of potentially effective and affordable medical treatments such as repurposed drugs. Our focus is on repurposed drugs — FDA approved drugs or therapies developed to treat one condition that could provide life-saving treatment against another disease like cancer or COVID-19.

Dear Morningside brainstorm team, below are 3 concepts based on the co-design sessions we held with physicians providers that aim to ***increase participation*** *in repurposed drug trials and* ***increase prescriptions.***

Please rank the below 3 concepts by sliding them in order of which you think would most influence your participation in repurposed drug clinical trials and repurposed drug prescriptions.

CONCEPT: Morningside Fellowship Program (Would you consider becoming a Morningside Fellow?)

A designation that identifies physicians who are involved in repurposed drug clinical trials or have knowledge/experience with particular repurposed drugs. Fellows are promoted on Morningside website, encouraged to present their repurposed drug research at meetings, receive promotional support and funding.

CONCEPT: Repurposed Clinical Trial Support Package (Would you consider starting a repurposed clinical trial?)

The clinical trial support package includes creating a regulatory binder, IRB submission, administrative tasks, and support for recruiting.

CONCEPT: Repurposed drug Digital Resource/Database (Would you contribute to and engage with this database and repurposed drug clinical trial PIs?)

A database of repurposed drug publications and clinical trial information sorted based on condition. Information regularly updated and local repurposed drug trials would have PI contact info available for inquiries.

How could these ideas work better for you? Please provide thoughts on ways these concepts could further increase your interest and support of repurposed drug options for patients.

→

Repurposed Clinical Trial Support Package

Concept	Physican Insights
The **clinical trial support package** includes creating a regulatory binder, IRB submission, administrative tasks and support for recruiting.	I'm extremely busy and I need help if I’m going to conduct a clinical trial
	The administrative burden of clinical trials is too much for me to manage
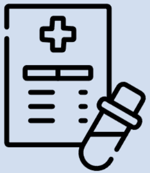	OLD trial enrollment is 10x more difficult;<br>I need more enrollment support

Morningside Fellows – Physican Influencers

Concept	Physican Insights
The **Morningside Fellowship** designation identifies physicians who are involved in repurposed drug clinical trials or have knowledge/experience with repurposed drugs. Fellows are promoted on Morningside website, encouraged to present their repurposed drug research at meetings and receive promotional support and funding. 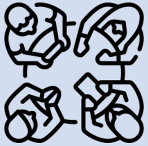	I learn from and seek information from my peers
	I'm likely to consider OLDs if I see physicians that I respect using them
	I want to hear about OLDs verbally (in meetings, calls, in person)

Morningside Digital Resource/Database

Concept	Physican Insights
A **database of repurposed drug publications** and clinical trial information sorted based on Condition. Information regularly updated and local repurposed drug trials would have PI contact info available for inquiries.	I want to know who is working on off-label drugs clinical trials
	I don't really know what the best resources are for finding information on off-label drugs
	I trust people in my network if I have questions about off-label drugs

Concept Vote Status (7/8)

"Please rank the 3 concepts in order of which you think would most influence your participation in repurposed drug clinical trials and repurposed drug prescriptions."

Concept	Rank	Minimum	Maximum	Mean	Std Deviation	Variance	Count
Morningside Fellows	**3**	1.00	3.00	2.00	0.93	0.86	7
Repurposed Clinical Trial Support Package	**3**	1.00	3.00	1.71	0.70	0.49	7
Morningside Digital Resource/Database	**1**	1.00	3.00	2.29	0.70	0.49	7
